# Endocuff Vision-Assisted Resection for Difficult Colonic Lesions—Preliminary Results of a Multicenter, Prospective Randomized Pilot Study

**DOI:** 10.3390/jcm12154980

**Published:** 2023-07-28

**Authors:** Rossella Palma, Gianluca Andrisani, Gianfranco Fanello, Augusto Lauro, Cristina Panetta, Chiara Eberspacher, Francesco Maria Di Matteo, Samuele Vaccari, Noemi Zorzetti, Vito D’Andrea, Stefano Pontone

**Affiliations:** 1 Department of Surgery, “Sapienza” University of Rome, 00161 Rome, Italy; rossellapalma89@gmail.com (R.P.); augustola@yahoo.com (A.L.); cristina.panetta@uniroma1.it (C.P.); chiara.eberspacher@gmail.com (C.E.); samuelevaccari@gmail.com (S.V.); noemi.zorzetti@gmail.com (N.Z.); vito.dandrea@uniroma1.it (V.D.); 2 Digestive Endoscopy Unit, Campus Bio-Medico, University of Rome, 00185 Rome, Italy; gianluca.andrisani@gmail.com (G.A.); f.dimatteo@unicampus.it (F.M.D.M.); 3 UOC Gastroenterologia ed Endoscopia Digestiva, Azienda Ospedaliera “San Giovanni-Addolorata”, 00184 Rome, Italy; gianfranco.fanello@libero.it

**Keywords:** stability, SMSA, mucosectomy, shaking, colorectal cancer, colonoscopy, adenoma

## Abstract

Background—Screening programs for colorectal cancer are implemented due to their ability to reduce mortality. The Endocuff Vision is a new endoscopic device that significantly improves the adenoma detection rate. The primary outcome was to assess the efficacy of ECV in improving stability and reducing operation time during difficult colon polypectomies in a multicenter randomized prospective study. Methods—In a randomized multicenter pilot study, two groups of patients who underwent difficult polypectomies with and without the assistance of Endocuff Vision were compared. Demographics and clinical characteristics of patients were obtained, and polyps’ size, morphology, site, and access (SMSA); polypectomy time; and endoscope stability were evaluated. Results—From October 2016 to April 2020, 32 patients were enrolled. In total, 12 patients underwent Endocuff Vision polypectomy, and 20 patients underwent standard polypectomy by using a computer-generated random number table. No statistical differences were found in clinical characteristics, SMSA, and polypectomy time. The most interesting findings were the positive correlations between shaking and SMSA (r = 0.55, *p* = 0.005) and shaking and polypectomy time (r = 0.745, *p* < 0.0001). Conclusion—Endocuff Vision seems to be adequately stable during difficult endoscopic resection procedures. The new parameter proposed that shaking is strongly correlated to the stability of the endoscope, the difficulty of the resection (SMSA), and the polypectomy time.

## 1. Introduction

Colorectal cancer (CRC) is the second most common tumor in women and the third most common in men and accounts for 10% of all types of tumors worldwide [1,2]. Screening programs for CRC are currently implemented because randomized trials have documented an association between screening and a sustained reduction in colorectal cancer mortality [3]; however, the effectiveness of colonoscopy is strongly associated with its quality. The most used quality indicator is the adenoma detection rate (ADR). Low ADRs correlate with higher post-colonoscopy colorectal cancer (PCCRC) rates and poorer outcomes [4]. Measures to improve ADRs have been developed. Lesions located on the proximal side of colonic folds pose a problem, and established maneuvers, such as retroflexion, may not be possible in most regions of the colon. Hence, devices attached to the tip of the endoscope have been created to flatten folds, improving the ADR [5].

Polyp size, location, and morphology substantially influence the difficulty of endoscopic polypectomy [6]. Although there is no standard definition for difficult polyps, polyps longer than 20 mm in diameter, polyps that occupy at least two haustral folds or more than one-third of the colonic circumference, or polyps that are located in particular anatomic regions are considered difficult [7]. Approximately 10–15% of colonic polyps are difficult polyps [6].

**Figure 1:**
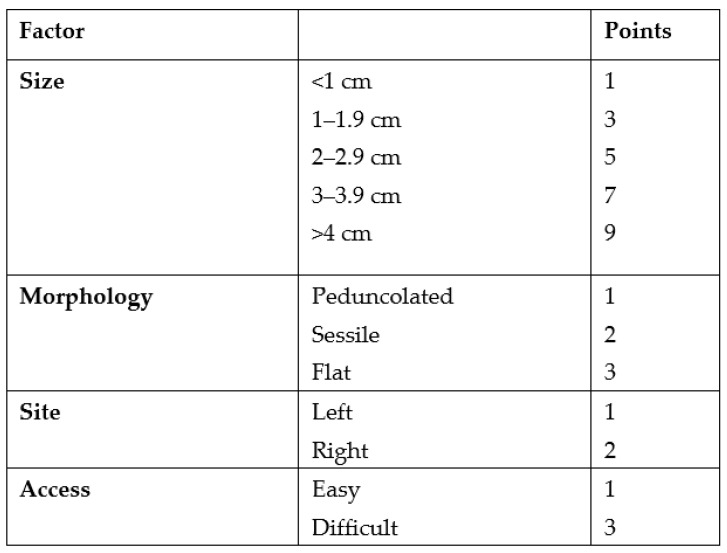
SMSA scoring system.

Size, morphology, site, access (SMSA) is a scoring system for the difficulty encountered during polypectomy (Figure 1) [8].

A multivariate regression analysis in the Munich Polypectomy Study (MUPS) revealed that polyp size is the main risk factor for complications. Right-sided polyps were a significant risk factor for major complications, and polyps larger than 10 mm in the right colon or 20 mm in the left colon and multiple polyps posed increased risks. A cut-off value of 3% was an acceptable rate for major complications [9].

The Endocuff Vision (ECV) is a new endoscopic device, equipped with eight flexible branches arranged in a single row, that is attached to the distal tip of the colonoscope. These branches are used to flatten the folds of the colon while withdrawing the colonoscope, facilitating improved visibility behind the folds. The use of an Endocuff significantly improves the ADR [10,11,12] and access for complex polypectomy and scar assessment in the sigmoid colon [13]. To date, no studies have investigated the efficacy of ECV in improving resection of difficult colonic lesions.

The primary outcome of this pilot study was to assess the efficacy of ECV in improving stability and reducing operation time during difficult colon polypectomies in a multicenter randomized prospective study.

## 2. Materials and Methods

This multicenter randomized prospective study included two groups of patients: patients who underwent standard polypectomy (SP) or ECV-assisted polypectomy (EP) for the resection of difficult polyps. This study was approved by the Sapienza University of Rome (protocol number: RP120172B89B515C). Block randomization of the two groups was performed using computerized randomization lists. Patients who were at least 18 years old were considered eligible. This study was performed in accordance with the ethical standards as laid down in the 1975 Declaration of Helsinki (2013 revision).

The exclusion criteria were: known colonic strictures; acute diverticulitis within six weeks before the examination; acute exacerbation of chronic inflammatory bowel disease; pregnancy; and the inability to give informed consent.

The data were collected from three Italian centers, and there were a different number of procedures for each center. Three expert endoscopists (SP, GA, and GF) performed the procedures, and all the data were analyzed by the same external medical observer (RP). Informed consent was obtained from all individual participants included in the study, and a video recording was made during each procedure. The overall required sample size was calculated.

Colon cleansing was obtained using split-dose low volume (2 liters) bowel preparation. The exams were performed under deep or conscious sedation based on the availability of sedatives at each center. Initial assessments were performed with conventional colonoscopes and without endoscopic accessories. The colonoscopy started with the patient lying in the left lateral position, and a complete colonoscopy without the ECV was performed. If endoscopic access was difficult and one or more complex polyps were observed, ECV was placed randomly by using a computer-generated random number table. In patients in which a difficult polyp was already diagnosed, the colonoscope was pushed straight forward to the site of the polypectomy. Overall withdrawal and procedure times were recorded. The “shaking” was calculated as the number of failed attempts to maintain the subject (the lesion) in the correct position of the framework while performing the polypectomy (it was usually placed at the right lower corner of visual field). All the procedures were recorded and analyzed by the same external medical observer. Procedural complications, such as bleeding and perforation, were recorded. The Paris classification of early and/or superficial tumors in the GI tract was used based on the morphology of the polyps. The LST classification was used in addition to the Paris classification to stratify these larger lesions based on their risk of invasive growth [14]. The external medical observer (RP) conducted further case selection by reviewing video recorded during the proceedings. All cases where the required SMSA score was not achieved were excluded.

## 3. Definitions

In order to assess the significance of the data, both standardized and original definitions were used.

- Difficult polyp: ≥8 score according to the SMSA scoring system.
- Lifting sign: separation of the lesion from the muscularis propria and lifting in response to submucosal injection [15].
- Procedure time: begins with the insertion of the colonoscope, including therapeutic interventions, and ends with the removal of the endoscope.
- Polypectomy time: begins with the submucosal infiltration and ends with retrieval of the polyps.
- Shaking: the number of attempts to maintain the right position of the scope with the subject in the center of the field of view during a polypectomy.
- Withdrawal time: begins with the withdrawal of the colonoscope from the cecal pole, excluding the time spent on interventions, and ends with the removal of the endoscope.

### Statistical Analysis

Dichotomous variables, expressed as numbers and percentages, were compared with chi-square and logistic regression models. One-way ANOVA was used to compare continues variables, expressed as mean ± standard deviation. A multivariate logistic regression analysis was used to evaluate the factors influencing polypectomy time. *p*-values < 0.05 were considered significant. Stata 15.0 was used for the analyses.

## 4. Results

From October 2016 to April 2020, 32 patients were enrolled (male: 13; mean age: 69). In total, 12 patients underwent EP, and 20 patients underwent SP.

The distribution of enrolled patients in the different centers was:

- Centre 1 = 8 (EP); 14 (SP);
- Centre 2 = 3 (EP); 5 (SP);
- Centre 3 = 1 (EP); 1 (SP).

Three patients in the EP group had two different difficult polyps, and one patient in the SP group was evaluated to have two difficult polyps with a total of 37 lesions. Demographic and clinical characteristics are described in Table 1. The main characteristics of the polyps are represented in Table 2. Most polyps presented a positive lifting sign (19 for the SP and 12 for the EP). The cecum intubation rate was 90% for the SP group and 50% for the EP group. Some patients did not need a complete endoscopic examination because they had already undergone diagnostic colonoscopies and in these patients the colonoscopy was performed up to the polypectomy site.

**Figure 2:**
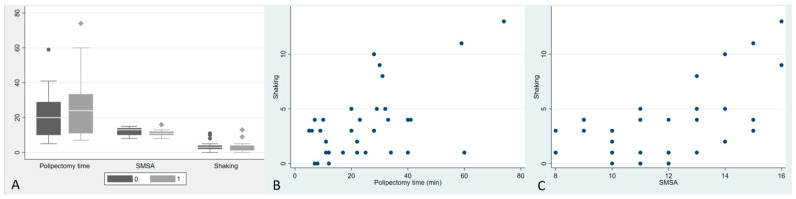
(**A**) Statistical comparison between the two study groups (SP = 0; EP = 1) on polypectomy time, SMSA and Shaking; (**B**) Correlation between Shaking and Polypectomy time; (**C**) Correlation between Shaking and SMSA. Bullets in B and C represent the graphic positioning of the single exams based on Shaking and Polipectomy time (**B**) or SMSA (**C**).

Procedural characteristics are shown in Table 3. No statistical differences were found in polypectomy time (Figure 2A). The maximum polypectomy time was 74 min in a patient who underwent EP and had an LST-granular type of cecum.

The mean shaking was calculated as 3.8 attempts in the SP group (range: 0–11) and 3.2 (range: 0–13) for the EP group (*p* = ns). The maximum number of failed attempts to maintain the right position of the scope during polypectomy was recorded in a 70 mm LST-GT section of the cecum.

There were no statistically significant differences in SMSA values between the two groups. The mean SMSA value was 12.1 ± 2.3 (range = 8–15) for the SP group and 11.1 ± 2.4 (range = 8–16) for the EP group (Figure 2A).

There was a positive correlation between shaking and SMSA values (r = 0.496, *p* = 0.004) and polypectomy time (r = 0.447, *p* = 0.008) (Figure 2B,C).

One patient in the SP group experienced bleeding, which was controlled endoscopically with argon plasma coagulation, on the fifth day post-polypectomy. One patient in the SP group underwent right hemicolectomy due to a late perforation in the polypectomy site.

The analysis was successively restricted to polyps ≥ 20 mm. In total, 16 lesions were observed in the SP group and 10 in the EP group. There were no statistically significant differences in polyp size.

The mean SMSA values were 12.9 ± 1.9 for the SP group and 11.6 ±1.9 for the EP group (*p* = 0.09).

The polypectomy took 22,5 ± 14.3 min for the SP group and 18.2 ± 9.2 min for the EP group (*p* = 0.171).

The mean shaking was calculated as 4.1 ± 3.2 attempts for the SP group and 2.7 ± 2.9 attempts for the EP group (*p* = ns).

There were positive correlations between shaking and SMSA (r = 0.55, *p* = 0.005) and between shaking and polypectomy time (r = 0.745, *p* < 0.0001).

The univariate regression analysis, which was adjusted for age and gender, demonstrated that shaking was influenced by dimension and SMSA. In particular, a 1 mm increase in polyp size causes a 0.1 increase in shaking (*p* = 0.05), while an increase of 1 SMSA value causes a 0.7 increase in shaking (*p* = 0.003). Moreover, shaking significantly influenced the polypectomy time: an increase of 1 in shaking causes a 2.8 min increase in polypectomy time.

A multivariate regression analysis, aimed at evaluating factors influencing polypectomy time, showed that, among SMSA, size, and shaking, shaking was the only significant determinant of polypectomy time (coefficient: 2.5, *p* = 0.017).

## 5. Discussion

The detection and removal of adenomatous polyps during colonoscopy prevents the development of CRC. The technical difficulties associated with these procedures can increase depending on the location and size of the lesions. The Endocuff was developed primarily to improve the detection of lesions located in these difficult locations [12,16]. ECV is a second-generation device that replaced the original Endocuff, which had two rows of shorter, firmer projections. The main aim of the Endocuff is to improve ADRs, and this has been investigated in several studies [11,17,18,19,20,21,22,23].

The ECV facilitates colonoscopic access for complex polypectomy and scar assessment in the sigmoid colon. A flat elevated-type lesion of the hepatic flexure, which was only detected using retroflexion, was successfully treated by underwater polypectomy using the Endocuff [24].

In a recent trial, von Figura et al. [25] investigated the ability of the ECV to reduce polypectomy time. The hypothesis was that cap assistance would ease polyp removal, which would result in shorter polyp removal and procedure times. There was a significant reduction in polypectomy time when the ECV was used compared to when it was not used (*p* = 0.02). Cap devices are useful in facilitating polyp resection but a limitation of this trial was the heterogeneity with respect to polyp size and polyp resection technique. Furthermore, the available data regarding the use of ECV during polypectomy are limited to complex polyps that could potentially be removed using a cap that holds down folds and improves visualizations [13].

We previously assessed the efficacy of ECV during difficult colon polypectomies according to the SMSA scoring system [26]. In our previous preliminary results, stability was significantly higher for ECV-assisted polypectomy (*p* = 0.025). This finding was not confirmed in this study.

The main limitation of this study is the limited sample size. Another limitation is its non-blinded design because the flexible arms of the device were necessarily visible.

Furthermore the cecum intubation rate was very different between the two groups. In fact, some patients did not need a complete colonoscopy and this represents another important bias.

**Figure 3:**
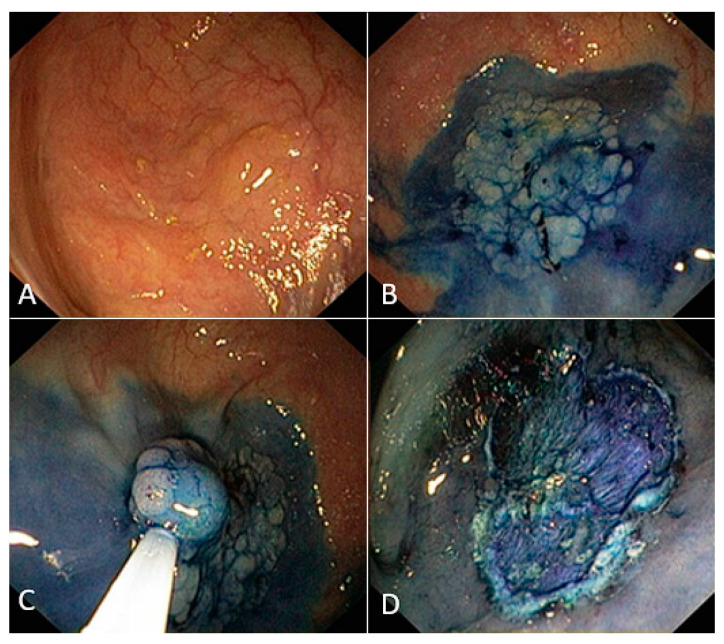
A difficult flat colonic lesion in the Standard Polypectomy Group. (**A**) White light visualization; (**B**) visualization after infiltration with vital dye; (**C**) piece-meal resection; and (**D**) final result.

Additionally, it is difficult to give an objective definition of stability. To define stability, we imagined the endoscopists looking for a “static shot” during the operations. Similar to a movie director, the operator precisely delimits the location of the subject of the shooting while everything else is considered “off screen”. In our case, the colonic lesion represents the subject of the framework and the surrounding elements represent the co-subjects and the background. As an example, a diverticulum close to the lesion is a co-subject. The co-subject may divert the operator’s attention from the subject of the shot. The background of the image is the normal colonic mucosa, which does not distract the operator (Figure 3).

In the field of photography, frames are defined as “static” when the field of view is constant and the camera does not change its aim.

Following this logic, we defined the image stability as the ability to keep the subject exactly in the center of the screen. We computed the stability by recording all the movements that the operator makes to reposition the “subject” in the center of the field of view, thus obtaining an indirect measure defined as shaking.

The most interesting findings were the positive correlations between shaking and SMSA (r = 0.55, *p* = 0.005) and shaking and polypectomy time (r = 0.745, *p* < 0.0001).

The univariate regression analysis showed that shaking was influenced by size and SMSA, and that this indirect parameter of stability significantly influenced the polypectomy time (coefficient: 2.5, *p* = 0.017).

The ECV may facilitate the withdrawal of the scope and polyp removal by stabilizing the position of the colonoscope, but there is no a validated evidence.

The number of patients should be increased to evaluate the real effectiveness of ECV in reducing the intervention time and the complication rate during difficult colon polypectomies. There is also a need for adequate technological support using digital tools to provide an accurate definition of stability. Despite the limited sample size, the preliminary results of this study are encouraging. The use of the ECV seems to be related to the adequate stability of the endoscope during difficult endoscopic resection procedures. The new parameter proposed that shaking is strongly correlated to the stability of the endoscope, the difficulty level of the resection, and the polypectomy time.

Although there is a need for larger randomized trials, the ECV is a handy device, which is easy to use and can facilitate the endoscopy of difficult colonic lesions, particularly polyps greater than 20 mm in diameter or localized in difficult areas, reducing the procedure time. The ECV represents a topic that undoubtedly deserves studies with a higher sample size in order to be able to establish whether there is actually an advantage for “difficult” procedures. Our strongest speculation is centered on the new stability metric: the shaking. Despite the limited sample size, we strongly correlated shaking with SMSA score, lesion size, and procedure time, providing an interesting starting point for future studies.

## Figures and Tables

**Table 1:** Demographics and clinical characteristics.

	SP	EP	*p*-Value
Patients, N	20	12	
Median Age, y	68.4 ± 10.5	70.1 ± 8.1	ns
Males (%)	11 (55)	2 (16.7)	0.033
First colonoscopy (%)	17 (85)	6 (50)	ns
Diabetes (%)	5 (25)	1 (8.3)	ns
Hypertension (%)	10 (50)	4 (33.3)	ns
Acetylsalicylic acid (ASA) (%)	3 (15)	2 (16.7)	ns
Diverticulosis (%)	12 (60)	6 (50)	ns

**Table 2:** Polyps main characteristics.

Variable	SP	EP	*p*-Value
Polyps number	23	14	
Polyp size in mm	29.8 ± 17.3	24.5 ± 17.8	ns
Polyp location	1R, 3 S, 2 D, 1 T, 7A, 7C	1 R, 6 S, 1 D, 2 T, 2 A, 2 C	
Peridiverticular polyp (%)	2 (9.5)	3 (18.7)	ns

Polyps location = (R: rectum, S: sigmoid, D: descending, T: Transverse, A: ascending, C: cecum).

**Table 3:** Outcomes of the endoscopic procedures.

Variable	SP	EP	*p*-Value
Polypectomy time *	25.4 ± 14.3	30.8 ± 19.8	ns
Procedure time *	37.7 ± 14.1	52.2 ± 28.7	<0.05
Withdrawal time in min *	6.1 ± 5.2	9.8 ± 9.5	ns
Bleeding	4 (20)	2 (16.7)	ns
Perforation	1 (5%)	0	ns
Shaking	3.8 ± 2.9	3.1 ± 3.9	ns

Polypectomy time was expressed in minutes. Data were expressed as number (%) or * mean ± SD.

## Data Availability

No data is available.

## Abbreviations

CRC	colorectal cancer;
ADR	adenoma detection rate;
PCCRC	post-colonoscopy colorectal cancer;
SMSA	size: morphology, site, access
MUPS	Munich Polypectomy Study;
ECV	Endocuff Vision:
SP	standard polypectomy;
EP	ECV-assisted polypectomy;
GI	gastro-intestinal:
LST	laterally spreading type;
GT	granular type:
